# Comparison of lens- and fiber-coupled CCD detectors for X-ray computed tomography

**DOI:** 10.1107/S0909049510044523

**Published:** 2010-12-02

**Authors:** K. Uesugi, M. Hoshino, N. Yagi

**Affiliations:** a Japan Synchrotron Radiation Research Institute, 1-1-1 Kouto, Sayo-gun, Sayo-cho, Hyogo 679-5198, Japan

**Keywords:** X-ray imaging, fiber-coupled CCD, lens-coupled CCD, microtomography

## Abstract

X-ray imaging detectors with an identical phosphor and a CCD chip but employing lens- and fiber-coupling between them have been compared. These are designed for X-ray imaging experiments, especially computed tomography, at the medium-length beamline at the SPring-8 synchrotron radiation facility. It was found that the transmittance of light to the CCD is about four times higher in the fiber-coupled detector. The uniformity of response in the lens-coupled detector has a global shading of up to 40%, while pixel-to-pixel variation owing to a chicken-wire pattern was dominant in the fiber-coupled detector. Apart from the higher transmittance, the fiber-coupled detector has a few characteristics that require attention when it is used for computed tomography, which are browning of the fiber, discontinuity in the image, image distortion, and dark spots in the chicken-wire pattern. Thus, it is most suitable for high-speed tomography of samples that tend to deform, for example biological and soft materials.

## Abstract 2

Lens- and fiber-coupled X-ray detectors with identical CCD chips were compared in their performance in high-resolution computed tomography experiments.

## 1. Introduction

It is common to construct an area X-ray detector with a scintillator, which converts X-rays to visible light, and a camera to observe the scintillation. For the camera, TV cameras were used in early models but now charge-coupled-device (CCD) cameras are the most common (Gruner, 1989 ▶; Tate *et al.*, 1997 ▶; Gruner *et al.*, 2002 ▶). The capabilities of the CCD such as its good linearity, fast readout and high sensitivity make it a competitive X-ray detector. In experiments at synchrotron radiation facilities, such detectors are widely used for diffraction and high-resolution imaging studies.

Since the CCD chips are generally smaller than the required field of view, it is often necessary to employ a coupling system to reduce the image size. The problems associated with the coupling between the scintillator and the CCD have been discussed in various fields of X-ray imaging for many years (Liu *et al.*, 1994 ▶; Tate *et al.*, 1997 ▶). The most commonly used coupling techniques are lens- and fiber-couplings, both of which have been used for more than 20 years. Many fiber-coupled detectors with tapered fibers have been developed for crystallography (Phillips *et al.*, 2002 ▶; Suzuki *et al.*, 1999 ▶). The lens-coupled detectors have been developed not only for high-resolution imaging (Pahl, 1995 ▶; Koch *et al.*, 1998 ▶) but also for diffraction experiments (Tate *et al.*, 2005 ▶; Madden *et al.*, 2006 ▶).

The fiber- and lens-couplings have their particular advantages and disadvantages. Although there is a general understanding that the fiber-coupling is more efficient (brighter) than the lens-coupling, the lens-coupling is still widely used for computed tomography (CT) at synchrotron radiation facilities. This is primarily because an accurate comparison using the same scintillator and the same CCD in both detectors has not been made so far. At the SPring-8 third-generation synchrotron radiation facility, phosphor-CCD detectors with lens-coupling have been developed in collaboration with Hamamatsu Photonics KK for high-resolution imaging experiments (Uesugi *et al.*, 2001 ▶). They are used for beam diagnostics at experimental hutches of about 20 beamlines and also in imaging experiments at BL20B2 (Lewis *et al.*, 2005 ▶), BL20XU (Parsons *et al.*, 2008 ▶), BL47XU (Nakamura *et al.*, 2008 ▶), BL40XU (Uesugi *et al.*, 2006 ▶) and BL04B1 (Funakoshi *et al.*, 2002 ▶). Among these applications, some CT experiments are carried out at a moderate spatial resolution of up to a few micrometers that can also be achieved by fiber-coupling. At BL20B2, an X-ray beam with a cross section of 300 mm (width) by up to 30 mm (height) is available. Experiments using such a large beam have been performed so far with a detector equipped with a large lens (‘Beam Monitor 5’) which is characterized below. Recently, for comparison with such lens-coupled detectors, a fiber-coupled detector has been developed. Since this detector uses the same scintillator and CCD camera as the Beam Monitor 5, a direct comparison of the coupling methods can be made. Here we investigate the merits of the two types of detectors in micro-CT experiments.

## 2. Materials and methods

### 2.1. X-ray source

The experiments were carried out at the BL20B2 ‘medium-length’ beamline at the SPring-8 synchrotron radiation facility (Hyogo, Japan) (Goto *et al.*, 2001 ▶). The beamline is equipped with a Si(111) double-crystal monochromator. The experiments were performed in the third hutch at 207 m from the X-ray source which is a bending magnet of the synchrotron. For most of the tests an X-ray energy of 15–25 keV was used. The X-ray flux was measured with an air-filled ionization chamber (S-1194, OKEN; Tokyo, Japan) in the first hutch (at about 45 m from the source). Most of the beam path was evacuated to avoid absorption by air.

### 2.2. Lens-coupled detector

**Figure 1:**
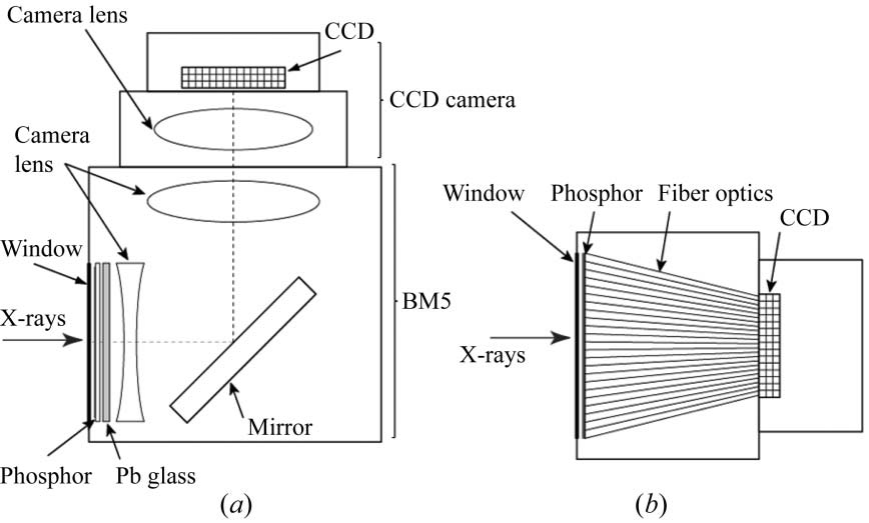
Design of the CCD detectors. (*a*) Lens-coupled detector. The X-ray beam enters through the carbon window and creates light in the phosphor. The image on the phosphor is viewed through a tandem lens after being reflected by a mirror. The first half of the tandem lens is housed within the X-ray detector (BM5) and the second half attached to the CCD camera with a PENTAX 67 mount. The camera and BM5 are coupled with a Philips mount. (*b*) Fiber-coupled detector. The phosphor is directly deposited on the tapered optical fiber. The CCD is also directly bonded to the fiber.

The characteristics of the two detectors are summarized in Table 1 ▶ and schematic drawings of their designs are shown in Fig. 1 ▶.

The X-ray detector with lens-coupled system evaluated in this study has been used over the last ten years at SPring-8. It was manufactured by Hamamatsu Photonics KK (Hamamatsu, Japan) and called ‘Beam Monitor 5’ (BM5). Its design is based on the optical unit of an image intensifier for medical imaging (Fig. 1*a* ▶). The phosphor is GADOX (P43, Gd_2_O_2_S:Tb^+^) powder deposited on a 5 mm-thick quartz plate and covered by a thin aluminium layer. The condensation method was used to form a thin layer of GADOX (Gruner *et al.*, 1993 ▶). The efficiency (absorption) of GADOX was calculated using the mass attenuation coefficient (with a linear interpolation) and density from the NIST database (http://www.nist.gov/pml/data/xraycoef/index.cfm). The phosphor thickness can be chosen depending on the purpose of the imaging experiment. We usually use 15 µm-, 25 µm- and 50 µm-thick phosphors for 10–80 keV X-rays. The choice is made according to the X-ray energy (which affects absorption by GADOX) and the required spatial resolution. There is a remote-controlled mechanism to move the phosphor along the incoming X-rays to adjust the focus. A plate of glassy carbon (1 mm-thick) is used to shield the ambient light. Just behind the phosphor are a lead-glass plate and a concave lens. A mirror is placed behind so that the large lenses and the CCD camera are not in line with the X-ray beam. This is a necessary precaution for a detector that is used with high-energy (up to 120 keV) X-rays. The two lenses in BM5 are from Chinon Corporation (Nagano, Japan). The concave lens behind the phosphor, which is necessary to reduce aberration, and the compound lens after the mirror give a focal distance (*f*) of 200 mm and the ratio of the focal length to the effective diameter (*F*#) is 1.65.

The camera used in combination with BM5 was a Hamamatsu Photonics C9300-124S with a Kodak KAI-11002M chip. The characteristics of the chip are shown in Table 2 ▶. The full-well depth is 40000 electrons which is divided into 12 bits, thus each analog-to-digital converter (ADC) unit corresponds to 10 electrons. The chip was cooled to 283 K, with a rather high dark current (21 electrons per second, *i.e.* 2.1 ADC s^−1^). However, since the exposure time is typically short (<1 s), the dark current is not a concern in actual use. The camera lens is an SMC PENTAX 67 (HOYA, Tokyo, Japan) with *f* = 105 mm and *F*# = 2.4.

### 2.3. Fiber-coupled detector

The detector (C9300-124F) manufactured by Hamamatsu Photonics KK is based on the Kodak KAI-11002M chip, which is also used in the lens-coupled detector. A tapered bundle of glass fibers (INCOM Inc., Charlton, MA, USA) with a demagnification ratio of 1.8:1 is directly bonded to the CCD chip. The phosphor is directly deposited on the wider end of the fiber. The thickness of the phosphor is 20 µm, which is covered with a thin aluminium layer. The window material is black paper. Since the CCD chip is cooled only to 263 K, the difficulties associated with thermal insulation and expansion are mostly avoided.

## 3. Results

### 3.1. Quantum efficiency

#### 3.1.1. Lens-coupled detector

The conversion gain was measured using a 21 keV X-ray beam of size 20 mm × 20 mm. The X-ray flux was divided by integrated pixel values to obtain an overall conversion gain, which was found to be 0.12 ADC units per X-ray photon with the 25 µm phosphor. With the 15 µm phosphor, the overall conversion gain was 0.07 ADC units per X-ray photon, while it was 0.18 ADC units with the 50 µm phosphor. The differences are due to absorption of the X-rays by the phosphor. Since the absorption of 21 keV X-rays by 15 µm, 25 µm and 50 µm GADOX (packing ratio 0.6) is about 20%, 30% and 52%, respectively, the conversion gain is 0.4 ADC units per absorbed X-ray photon with all three phosphors. With the camera conversion gain of ten electrons per ADC unit (full-well capacity of 40000 electrons divided by 12 bits), four electrons are estimated to be produced by each X-ray photon. This means that electrons for 10000 X-ray photons can be accumulated in each pixel before the full-well capacity is reached.

In a lens-coupled system, the light capture efficiency (LCE) of the lens can be obtained by (Liu *et al.*, 1994 ▶)
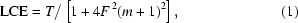
where *T* is the bulk transmission factor (typically 0.8 for each lens), *F* is the *F*-number of the lens and *m* is the demagnification factor. The demagnification factor is the ratio of the focal lengths of the two lenses, 200/105 = 1.90. According to Bien *et al.* (2007 ▶), the effective *F*-number of a tandem lens composed of lenses with *F*# of 1.65 and 2.4 can be calculated [their equation (16)] to be 1.57. Thus, the LCE is 0.0076.

The number of optical photons (*N*) per X-ray photon is
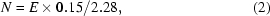
where 0.15 is the energy-conversion efficiency of GADOX (Gruner *et al.*, 2002 ▶) and 2.28 (eV) is the energy of a 545 nm photon. At 21 keV, 1300 photons are created by each X-ray photon, 0.0076 of which is ten optical photons. Because of the 48% quantum efficiency of the CCD for 545 nm, each optical photon is expected to create five electrons. This is a good agreement with the experimentally obtained value (four electrons).

#### 3.1.2. Fiber-coupled detector

The overall conversion gain of the CCD camera with a tapered fiber, obtained in the same manner as with a lens-coupled detector, was 0.43 ADC units (4.3 electrons) per X-ray photon. Since absorption of 21 keV X-rays by a 20 µm GADOX (packing ratio 0.6) is 25%, the conversion gain is calculated to be 1.7 ADC units, that is 17 electrons per absorbed 21 keV X-ray photon. Since the full-well capacity for 12 bits is 40000 electrons, this means that electrons for 2400 X-ray photons can be accumulated in each pixel. Since the readout noise of the camera, which was calculated from variation of a pixel value in successive dark frames, is 3.94 ADC (standard deviation), it is difficult to see each 21 keV X-ray photon.

The transmittance of a 1.8:1 tapered fiber is about 20% (Coleman, 1985 ▶). Because of the 48% quantum efficiency of the CCD, from the 1300 photons created by each absorbed 21 keV X-ray photon, the maximum number of electrons we should expect in the CCD is 125. The experimentally obtained value (17 electrons) is one-seventh of this. The unknown factors in the estimation are a loss of light within the phosphor, acceptance (numerical aperture) of the optical fiber, a loss of light within the fiber, and a loss owing to reflection at the fiber/CCD interface.

### 3.2. Uniformity of response

**Figure 2:**
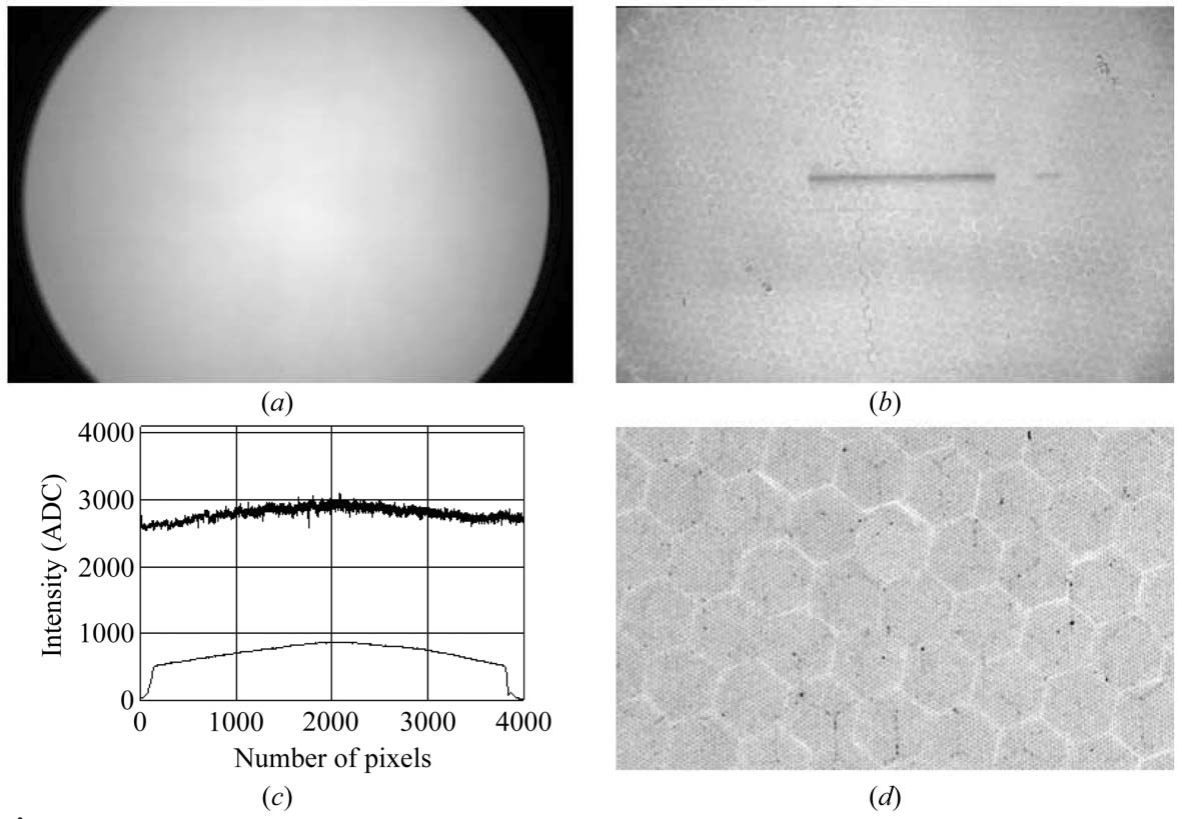
Uniformity of response. (*a*) A flat-field image recorded by the lens-coupled detector. The field of view is limited by the size of the phosphor, not by that of the CCD chip. (*b*) A flat-field image recorded by the fiber-coupled detector. The horizontally elongated area in the center with low intensity was caused by damage resulting from prolonged irradiation, most probably owing to browning of the optical fiber. These images were obtained with an X-ray energy of 23 keV. (*c*) Horizontal line profiles of the images at the center of images in (*a*) and (*b*). (*d*) Chicken-wire pattern in the fiber-coupled detector.

Since the vertical beam size at the BL20B2 beamline is smaller than the field of view of the detectors, the uniformity of response was studied by moving the detector vertically across the X-ray beam at a constant speed during an exposure. The uniformity of response in the entire area of view is quite specific to each detector (Fig. 2 ▶). In the lens-coupled detector, the shading of the lens system causes a global density gradient, brightest at the center with about 40% decrease at the periphery (Fig. 2*a* ▶). The smaller fluctuations (about 2% standard deviation) are seen at higher spatial frequencies, which are presumably due to the variation in the thickness of the phosphor. Since the grain size of the GADOX phosphor is about 1–3 µm, variation in the number of grains in the phosphor layer can cause a fluctuation in response to this magnitude.

In the fiber-coupled detector, the global gradient in the flat-field response (Fig. 2*b* ▶) is about 10%. This is probably due to the distortion in the fiber optics and can be corrected by software. The high-frequency pixel-to-pixel fluctuation in the response is about 5% in standard deviation. These variations are mostly caused by the so-called chicken-wire pattern of the fiber optics: the field of view is divided into hexagons with a diameter of about 100 pixels and, along the edges of the hexagons, the transmittance is either higher or lower compared with the area within the hexagon. Although not many, there are pixels with a large (up to 70%) drop in response (Fig. 2*d* ▶).

Noticeable non-uniformity can be caused by radiation in the fiber-coupled detector (Fig. 2*b* ▶). Continuous illumination with high flux density (less than 2 h exposure of 3 × 10^9^ photons mm^−2^ s^−1^ of 20 keV X-rays at the first hutch of BL20B2) created an area with low response. Since such a phenomenon has not been observed in the lens-coupled detector, it is most probably due to browning of the optical fiber. The 20 µm phosphor that was used in this experiment absorbs only about 30% of the 20 keV X-rays, while the rest is absorbed in the optical fiber and may have caused browning by creating color centers.

### 3.3. Linearity of response

The linearity of response is determined by the characteristics of the phosphor and the CCD, which are common between the two detectors. Thus, no significant difference is expected. Experimentally, with constant X-ray intensity, the output was linear to the exposure time in both detectors to the maximum well depth (|*r*| = 0.999998 and 0.999874 for the lens- and fiber-coupled detector, respectively).

### 3.4. Spatial resolution

**Figure 3:**
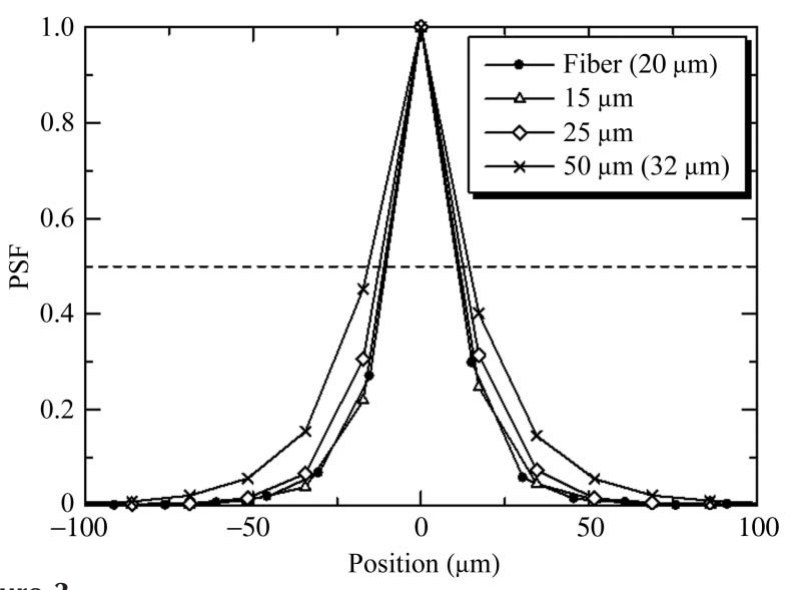
Spatial resolution. Point-spread functions (PSFs) obtained with a point beam. The pixel size for the lens-coupled and fiber-coupled detectors were 17.1 µm and 16.2 µm, respectively.

A point beam with a size of 5 µm (horizontal) × 6 µm (vertical) was created by cross slits. It was placed approximately at the center of a pixel and the point spread function (PSF) was measured (Fig. 3 ▶).

The rule of thumb is that the highest achievable spatial resolution of detectors using a phosphor is similar to its thickness (Gruner *et al.*, 1993 ▶). This tendency is confirmed in Fig. 3 ▶. The PSFs of the 15 µm and 25 µm phosphors are similar because the pixel size (17.1 µm) limits the resolution. The exception is the 50 µm phosphor in the lens-coupled detector, whose FWHM (full width at half-maximum) of the PSF is only less than 10 µm poorer than the 25 µm phosphor. However, the 50 µm phosphor has a much longer tail than the other thinner phosphors, showing that the scatter of light within the phosphor causes a serious spread in the PSF. Flare in a lens-coupled system has been found and discussed by Tate *et al.* (2005 ▶). Part of the tail in the plot of the PSF may be due to this. The PSF of the fiber-CCD is similar to that of the lens-coupled CCD with the similar thickness of phosphor.

### 3.5. Geometrical distortions

**Figure 4:**
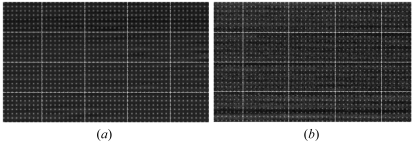
Geometrical distortion observed with a grid pattern (0.20 mm pitch). (*a*) Part of the grid pattern recorded with the lens-coupled detector. A small area at the upper-right corner of the view is shown. The lines are drawn for guidance. (*b*) Part of the grid pattern recorded with the fiber-coupled detector. An area at the center of the view is shown. The dots are aligned parallel to the left and right edges of the image but not to the top and bottom edges.

The geometrical distortion was measured using a grid pattern. The pattern was made in a 1 µm tantalum plate. The diameter and pitch of holes are 20 µm and 200 µm, respectively. In the lens-coupled detector it was difficult to detect the distortion (Fig. 4*a* ▶). Deviation from a line was found near the edge of the field but it was usually smaller than one pixel. The geometrical distortion of the fiber-coupled CCD was also measured using the grid pattern (Fig. 4*b* ▶). It can be seen that the dots are aligned parallel to the vertical edges of the image but not parallel to the horizontal edges. Thus, the vertical and horizontal axes of the dots are not orthogonal. However, this distortion is much smaller than previously reported (Suzuki *et al.*, 1999 ▶). Since these features are fixed and do not change with time, in principle, they can be corrected by software (Barna *et al.*, 1999 ▶).

### 3.6. Other characteristics

**Figure 5:**
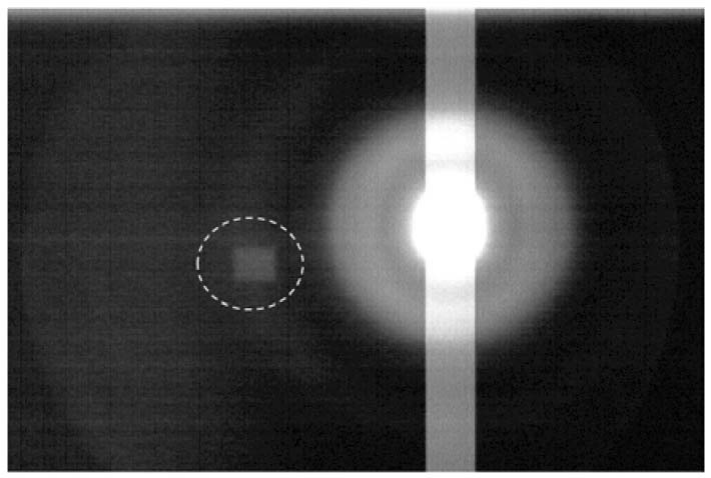
Stray light observed in the lens-coupled detector. A rectangular beam that was beyond the saturation level of the CCD camera was recorded to observe reflections and scatterings in the lens system. The dashed circle indicates a ‘ghost’ of the strong beam. This image is shown on a logarithmic scale.

Stray light was observed in the lens-coupled detector (Fig. 5 ▶). Although the reflection in the lens is suppressed by the use of coatings, it does not remove all reflections and scatterings. A ‘ghost’ image or flare owing to stray light is apparent when a strong light is introduced into the lens system as in Fig. 5 ▶, but the level of its intensity is less than a few thousandths of the incident light. To avoid saturation, such a strong light is usually not introduced into the lens in actual experiments. However, when there is a large area with high brightness in the image, the reflections and scattering may cause a global increase in the background that cannot be removed by dark-image subtraction in imaging experiments.

A fiber optics has been reported to have ‘zingers’, which are localized noise spikes caused by the scintillation of radioactive impurities in the glass fiber. Although this has been found to be serious in some fiber-coupled detectors (Barna *et al.*, 1999 ▶), we did not see any zingers in this detector.

## 4. Discussion

In the current study, CCD detectors with lens coupling and fiber coupling were compared. These were designed for X-ray imaging with a field of view of about 50 mm × 30 mm and a moderate spatial resolution (10–20 µm). For these purposes, both detectors fulfil the requirements. It should be noted that the X-ray imaging detectors with resolution better than 1 µm have only been achieved by the lens-coupled system (Koch *et al.*, 1998 ▶; Uesugi *et al.*, 2001 ▶) because, at this resolution, the diameter of the optical fibers in the fused fiber-optic bundle is larger than the resolution. The present results confirm that the fiber-coupled detector is about four times more efficient than the lens-coupled detector in the light transmission. This is the major advantage of the fiber coupling. The higher transmission enables a fourfold reduction in the exposure time, leading to fourfold increase in the number of samples to be studied in the limited beam time at synchrotron facilities. It is especially advantageous when the sample is prone to deform with time: a gradual change in structure causes artifacts in the reconstructed CT images. For these reasons, fast data collection is always favored in CT experiments. Also, the radiation dose on the sample is lower with a shorter exposure time.

Compared with the lens coupling, the fiber coupling is a relatively new technique and thus has been a subject of investigation for some years. Davis & Elliott (2006 ▶) tested a fiber-coupled CCD for laboratory-based microtomography. They found that the scattering of light through the fiber cladding caused serious blurring of an image. We did not find a similar phenomenon with our detector. Rather, the lens-coupled detector tends to have a background owing to reflections and scatterings in the lenses (Fig. 5 ▶). However, considering the difficulty that Davis & Elliott (2006 ▶) found, the quality of the optical fiber seems important.

In the fiber-coupled detector, the experimentally observed number of electrons per each absorbed X-ray photon is much lower than expected by calculation. Although there are some unknown factors in the calculation, the seven times difference seems large. The conversion gain observed in this study for the fiber-coupled detector (17 electrons per absorbed 21 keV X-ray photon) is to be compared with those of other fiber-coupled detectors: 100 electrons at 12 keV (with a 1:1 optical fiber; Phillips *et al.*, 2002 ▶), 6 electrons at 12 keV [Mar165 with a 2.7:1 tapered fiber (MarReseach GmbH, Norderstedt, Germany)] or 0.9–4.6 electrons depending on the CCD at 5.9 keV (Tate *et al.*, 1997 ▶). Since the conversion gain of the fiber-coupled detector used in this study is generally lower than other detectors considering the X-ray energy, there may be room for improvement.

Both types of coupling suffer from geometrical distortion and non-uniformity of response. These two factors are related because the change in the pixel size by distortion also affects the response. The fiber coupling especially has the drawback of the chicken-wire pattern and browning of the fiber, while the lens coupling has a more pronounced shading. In actual imaging experiments, these can be corrected by using a flat-field image. However, the very dark pixels in the chicken-wire pattern have transmittance less than 30% compared with neighboring pixels. When the image is dark in this area, only a small amount of light can be transmitted to the CCD. Since the low level of light tends to suffer from higher noise and non-linearity, this can cause a ring artifact in the reconstructed image.

**Figure 6:**
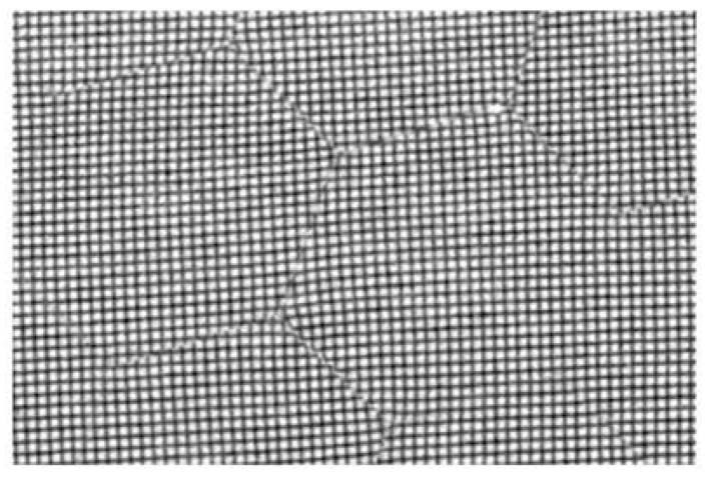
Distortion of a mesh pattern observed with a prototype fiber-coupled detector. A 750 Cu mesh (33.3 µm pitch) was imaged at 9 keV with a prototype fiber-CCD detector. A straight optic fiber (1:1) was used.

Another serious problem that may be caused by the chicken-wire pattern is discontinuity in the image. Fig. 6 ▶ shows an image of 750 mesh obtained with our prototype fiber-coupled detector which had a straight optical fiber. At the edge of the hexagons the mesh pattern is clearly discontinuous. Since some information on the object is missing, it is impossible to properly reconstruct an image when this type of image distortion occurs. Such discontinuity was not found in the fiber-coupled detector we tested in the present study. However, as no information should be lost in the process of image transmittance for CT, the absence of discontinuity needs to be carefully confirmed before employing a fiber-coupled detector. This is particularly important because most other applications of fiber-coupled detectors can be satisfactorily performed with a small amount of discontinuity.

Browning of the optical fiber has not been considered important in previous studies, because the fiber-coupled detectors are mainly used in diffraction experiments with low-energy X-rays. However, in imaging experiments higher energies tend to be used and the phosphor needs to be thin enough to achieve high spatial resolution, resulting in high transmission of X-rays to the tapered fiber. The lens-coupled detector may also have a browning problem but we have not found discernible damage either on the quartz substrate of the phosphor, the convex lens or the mirror. We do find browning of the lens when it is placed behind the phosphor without lead glass and this is the reason for the design of the detector employed here. Although browning can be dealt with by flat-field correction, a decrease in the transmission efficiency reduces the advantage of the fiber-coupled detector. As replacing the optical fiber is a major modification that can cost a large fraction of the price of the entire detector, attention should be paid to avoid excess radiation on a fiber-coupled detector. A replaceable faceplate may be used but it reduces the transmission efficiency because of the scatter at the interface between the optical fibers (Davis & Elliott, 2006 ▶).

From a practical point of view, it is often desired to change the camera in the detector. In a lens-coupled detector, a camera can be chosen and changed according to the requirement of each experiment. In particular, one important development in the imaging technology is CMOS (complementary metal oxide semiconductor) devices. CCDs are gradually being replaced by CMOS sensors in many fields of imaging. The major advantage of the CMOS camera is fast readout. For high-speed imaging, in lens-coupled detectors it suffices to replace a CCD camera with a CMOS camera, but a large-scale modification is necessary for a fiber-coupled detector. This is a point that needs attention in the practical choice of the detectors.

The high transmission of the fiber coupling is most useful when the exposure time (and hence the data collection time) and the X-ray dose on the sample needs to be minimized. Some soft and biological materials tend to deform during a long CT scan, and bubbles may appear in wet samples when the exposure dose is too high. It is difficult to keep live animals stably anesthetized during a long scan and high dose may affect their physiological condition. Also, real-time imaging often requires high speed that can be achieved more easily by efficient detectors. These are the experiments that benefit most from the use of fiber-coupled detectors. In other cases, we prefer the lens-coupled detector for CT because of its flexibility, robustness and ease of use.

## Figures and Tables

**Table 1:** Comparison of the detectors

	Beam Monitor 5 + C9300-124S (lens-coupled)	C9300-124F (fiber-coupled)
Lens *f*	200 and 105 mm	1.8:1 (taper ratio)
Lens *F*#	1.65 and 2.4	NA
Effective pixel size	17.1 µm	16.2 µm
CCD format	4000 × 2672	4000 × 2672
Field of view	64 mm × 45 mm	60 mm × 40 mm
Scintillator	GADOX (P43, Gd_2_O_2_S:Tb^+^)	GADOX
Scintillator thickness	15 µm, 25 µm, 50 µm	20 µm
Window material	Amorphous carbon (1 mm thick)	Black paper

**Table 2:** Characteristics of the CCD camera

Chip	Progressive scan interline CCD KAI-11002M
Effective number of pixels	4000 (H) × 2672 (V)
Pixel size	9.0 µm × 9.0 µm
Effective area	36.0 mm × 24.0 mm
Frame rate	Single tap: 2.5 Hz
	Dual tap: 4.5 Hz
Readout noise	Single tap: 40 electrons (typically)
A/D conversion	12 bits
Full-well capacity	40000 electrons
Quantum efficiency	48% (at 545 nm)
Contrast enhancement	0–14 dB
Cooling temperature	283 K (typically) at an ambient temperature of 293 K
